# Biosynthesis of silver nanoparticles with antibacterial, antioxidant, anti-inflammatory properties and their burn wound healing efficacy

**DOI:** 10.3389/fchem.2022.972534

**Published:** 2022-08-22

**Authors:** Bum-Erdene Bold, Enerelt Urnukhsaikhan, Tsogbadrakh Mishig-Ochir

**Affiliations:** ^1^ Laboratory of Molecular and Cellular Biophysics, Department of Biology, National University of Mongolia, Ulaanbaatar, Mongolia; ^2^ Graduate School of National University of Mongolia, Ulaanbaatar, Mongolia

**Keywords:** biosynthesis, silver nanoparticle, antibacterial activity, anti-inflammatory activity, anti-oxidant activity, wound ointment, burn injury

## Abstract

The current study aims to develop a novel burn wound ointment consisting of sheep’s tail ointment loaded with AgNP. The AgNP in the ointment serves as an antibacterial, antioxidant and anti-inflammatory agent. The AgNP was developed *via* the biological method with the assistance of the medicinal plant *Rhodiola rosea*. The characterization of AgNP was assessed using UV-Vis spectroscopy, FTIR, Zeta Potential, XRD, PCCS, SEM, and EDX techniques. The formation of AgNP was confirmed by UV-Vis spectrum at the absorbance of ∼430 nm, and the biomolecules responsible for reducing and capping the AgNP were characterized by FTIR analysis. The stability of AgNP was determined with Zeta potential, which revealed a highly stable colloidal solution with a surface charge of −68.38 ± 3.4 mV. The synthesized AgNP had a face-centered cubic structure with a crystallite size of 23 nm and average grain size of 67.5 nm. The SEM image showed a fairly monodisperse 20 nm-sized spherical-shaped AgNP. The synthesized AgNP contained high purity of the silver, and a low concentration of AgNP inhibited both Gram-positive and Gram-negative bacteria. Moreover, the scavenging activity of AgNP was investigated using DPPH and H_2_O_2_ scavenging assay, and the results revealed a dose-dependent antioxidant activity with the highest activity at a concentration of 450 μg/ml. Finally, the burn wound healing effect was evaluated by applying the AgNP-loaded ointment to the wound site of BALB/c mice. The *in-vivo* studies confirmed that AgNP-loaded ointment reduced the wound size, decreased the epidermis layer, and lowered mast cell migration compared to untreated burn wounds. And the synthesized AgNP regulated both pro-inflammatory and anti-inflammatory gene expression, thereby promoting burn wound closure on BALB/c mice. The developed AgNP-loaded ointment has the potential to be applied in the biomedical field.

## 1 Introduction

Burn injury is one of the leading public health concerns due to the severe injury sustained by the patient and the incurrence of increased treatment costs on dressing, medication, and surgery (Sharma et al., 2020). Based on the depth of the tissue injury, burn wounds can be classified into first to fourth degrees. And third or fourth-degree burn injuries are severe injuries requiring immediate surgical care (Lee and Jun 2019). Another major complication of burn injury is the infection of the wound, and it is considered one of the leading causes of mortality (Ahmed and Mustafa, 2020). The conventional method for wound healing involves transplanting healthy tissue to the site of injury as skin substitutes. However, the donor-site shortage, high risk of infection, and the tendency for scarring and contraction led to a rising interest in researching therapeutic agents with antimicrobial and anti-inflammatory properties (Banasiuk et al., 2020; Odeniyi et al., 2020).

Silver nanoparticles (AgNPs) are widely known for their antimicrobial, anti-cancerous, and anti-inflammatory effect (Sharma et al., 2020). The small size, high surface area, and dispersion rate are the main features that offer an antimicrobial effect with a long-lasting impact (Khan et al., 2020) and the penetration ability to microbial cells (Vikas et al., 2014). Synthesis of nanoparticles can be categorized into physical, chemical, and biological methods. The advantage of the biological method over other physical and chemical methods is its non-toxicity, cost-effectiveness, environmentally-safe approach (Naseem et al., 2020), and employment of natural capping and reducing agents (Shah et al., 2020). The biological method employs bacteria, fungi, algae, or plants to synthesize nanoparticles (Okaiyeto et al., 2021). The assistance of plants has gained wide attention due to no requirement for the maintenance and culture of microorganisms. Also, the plant species themselves have distinct bioactive compounds (Mohanta et al., 2020). The synthesis of nanoparticles through plants may include the use of extracts of flowers, stems, leaves, peels, latex, or roots (Rajput, Kumar, and Agrawal). In addition to these advantages, secondary plant metabolites with antioxidant effects may be more suitable for the biomedical field (Elgamouz et al., 2020). Medicinal plants are a rich source of polyphenols, flavonoids, tannins, coumarins, alkaloids, etc., (Javan bakht Dalir et al., 2020). These bioactive compounds are reported to reduce and stabilize nanoparticles (Wei et al., 2020). There are several reports on AgNPs synthesis *via* medicinal plants, such as *Carya illinoinensis* (Garibo et al., 2020), *Carduus crispus* (Enerelt et al., 2021), *Lysiloma acapulcensis* (Mahiuddin et al., 2020), *Caesalpinia pulcherrima* (Nouri et al., 2020), *Piper chaba* (Zarei et al., 2020), *Mentha aquatica* (Devi et al., 2020), *Caralluma tuberculata* (Alkhalaf et al., 2020), *Aegle marmelos* (Kambale et al., 2020), *Nigella sativa* (Carson et al., 2020), *Annona muricata* (Ansar et al., 2020), *Brillantaisia patula, Crossopteryx febrifuga*, and *Senna siamea* (Gavamukulya et al., 2020), etc. The stages of plant-assisted nanoparticle formation start by reducing metal ions from bivalent to zerovalent forms through the presence of metal salts and plant extract. The nanoparticles are formed from the coalescence of the reduced metal atoms (Pu et al., 2020).

In this study, we have synthesized AgNP with *Rhodiola rosea* root extract. *Rhodiola rosea*, also known as golden root, is from the plant family of Crassulaceae, recognized for their medicinal properties. *Rhodiola rosea* has various therapeutic effects such as anti-diabetic, anti-cancer, anti-aging, cardio-protective, and neuroprotective activity (Zhang et al., 2020). This plant has been used in traditional medicine in Asia (Shikov et al., 2020). Biologically active compounds in *Rhodiola rosea* were investigated; such as alkanols, benzyl, phenols, phenylethanes, gallic acid, phenylpropanoids, flavonoids, monoterpenoids, triterpenes, etc., (Bala and Rani, 2020). The present study aimed to develop AgNP-based ointment and study its effect on the burn wound murine model. We first synthesized AgNP with *Rhodiola rosea* and characterized their different properties, such as synthesis rate, yield, stability, crystallite size, and morphology. Next, we evaluated the antibacterial and antioxidant activity of AgNP. Finally, we formulated AgNP ointment and studied its anti-inflammatory effect on the burn wound murine model.

## 2 Materials and methods

### 2.1 Chemicals and preparation of AgNP ointment

The silver nitrate (AgNO_3_) with ≥99.0% purity was purchased from Sigma Aldrich. The Nutrient Broth and Nutrient Agar (HiMedia) were purchased from commercial sources and used without further purification. The sheep’s tail was purchased from the local market of Ulaanbaatar, Mongolia. The ointment was prepared according to the traditional method; in brief, the sheep’s tail was cut into small pieces and melted at a low temperature to obtain the absorption-based ointment. Afterward, the oil was filtered for further use in the study. 0.5% AgNP ointment was prepared by adding dry fine powder form of AgNP into sheep’s tail cream. All the other relevant reagents are up to standard.

### 2.2 Preparation of plant extract

The roots of *Rhodiola rosea* were washed with tap water to remove the adhering dust and soil particles, followed by washing with distilled water. The plant’s roots were cut into fine pieces for further study. Plant extracts were prepared by adding 100 ml of distilled water to 5 g of roots from *Rhodiola rosea*. Afterward, it was boiled for 15 min and cooled at room temperature, then centrifuged twice at 10,000 rpm/20 min. Finally, the plant extract is ready for the subsequent study.

### 2.3 Biosynthesis of AgNP

The plant extract of *Rhodiola rosea* was added to the 10^−3^M AgNO_3_ solution with a ratio of 1:32. The reaction took place at ambient temperature with direct exposure to sunlight. To obtain the dry powder form of AgNP, the nanoparticles were centrifuged three times at 18,000 rpm for 20 min and washed with deionized water. Afterward, pellets were kept inside the oven at a temperature of 400°C for 3 h.

### 2.4 Characterization of the synthesized AgNP

The color change from pale yellow to colloidal dark brown as a function of time indicates the presence of AgNP. The UV-Vis spectrophotometer (Shimadzu UV-2500PC Series) records the absorption spectra; the maximum absorbance of spectra shows the size and yield of the synthesized AgNP. The colloidal AgNP was measured at different time intervals from 5 min to 4 h within 300–700 nm wavelength. The biomolecules of the roots of *Rhodiola rosea* responsible for reducing silver ions and stabilizing AgNP were determined *via* FTIR spectrophotometer (Prestige-21, Shimadzu, Japan) operating within the range of 500–4000 cm^−1^ through the potassium bromide powder method. The Zeta Potential (ZetaCompact, CAD Instruments, France) was used to determine the surface charge of the synthesized AgNP. The size and shape of the synthesized silver nanoparticles were measured with Scanning Electron Microscope (ZEISS, Germany). Elemental analysis of the synthesized AgNP was performed on an Energy Dispersive X-ray spectroscope instrument (TM-10000 with EDX). The XRD analysis was employed to determine the average crystallite size of the synthesized AgNP. XRD (Shimadzu, Maxima-X-7000) with CuKα radiation and operating at 40 kV with a current of 30 mA in the range of 20°–90°. The average crystallite size of the synthesized AgNP was determined with the Scherrer equation. The particle size distribution of the biosynthesized AgNP was studied using Photon Cross-Correlation Spectrum (PCCS) on the NANOPHOX NX0137 instrument (Sympatec GmbH, Germany).

### 2.5 Antibacterial activity of the synthesized AgNP

The antibacterial activity of the synthesized AgNP with *Rhodiola rosea* (300 and 450 μg/ml) was assessed on *Staphylococcus aureus* and *Pseudomonas aeruginosa* using the agar well diffusion method. Standard antibiotics (Biolab ltd., Budapest, Hungary) including erythromycin 15 mcg/disc (*P. aeruginosa*) and penicillin G 6 mcg/disc (*S. aureus*), plant extract and HEPES buffer were selected as the positive and negative control group of the study. First, the bacteria were cultured in the liquid broth medium at 37°C until it reached OD_600_ = 0.6. Afterward, 100 µl of the respective bacterial suspension was spread on the agar plates. The samples and control groups were transferred and filled into the well on the agar plates, followed by incubation at 37°C for 24 h. Finally, the diameter of the inhibition zone around the wells was measured and analyzed.

### 2.6 Antioxidant activity of the synthesized AgNP

#### 2.6.1 DPPH free radical scavenging activity

1,1-diphenyl-2-picrylhydrazyl (DPPH) free scavenging assay was employed to determine the antioxidant activity of the synthesized AgNP (Bhakya et al., 2016). Different concentrations (10, 25, 50, 75, 150, 300, and 450 μg/ml) of AgNPs and Ascorbic acid were measured for the assay. Briefly, 1 ml of DPPH (1 mM) mixed with methanol was added to the above samples and vortexed thoroughly. Afterward, the mixture was incubated in the dark for 1 h at room temperature, the absorbance was measured at 517 nm using UV-Vis Spectrophotometer. Free radical scavenging activity was evaluated using the following equation:

Scavenging activity(%)=(Ac−As)/Ac×100 (1)

where *Ac* represents the control absorbance; *As* is the absorbance of DPPH in the AgNP solution at various concentrations/standard ascorbic acid.

#### 2.6.2 Hydrogen peroxide scavenging activity

The hydrogen peroxide (H_2_O_2_) assay was analyzed according to the method described by Keshari et al. (2020). Equal volumes (50 µl) of 5 mM H_2_O_2_ solution were mixed with different concentrations (10, 25, 50, 75, 150, 300, and 450 μg/ml) of AgNPs and ascorbic acid. Afterward, the solutions were incubated for 20 min at room temperature and the absorbance was measured at 610 nm. The percentage of (H_2_O_2_) scavenging was calculated Eq. 1.

## 3 *In vivo* evaluation of burn wound healing

### 3.1 Experimental animals

The animal study was reviewed and approved by the Institutional Animal Care and Use Committee of National University of Mongolia. BALB/c mice (*n* = 10) were housed in two separate cages with free access to water and standard rodent chow. The environment was controlled at 24–26°C under 12 h light/dark cycle. All mice were anesthetized with an intraperitoneal injection of ketamine (13 mg/kg) and xylazine (8 mg/kg) (Gomathi et al., 2017). Afterward, the dorsum area of the mice was swabbed with ethanol 70% solution before shaving.

### 3.2 Burn injury model and wound healing

Before inducing burn injury, BALB/c mice were randomly divided into two groups (*n* = 5 each): a control group and AgNP-ointment treatment group. Burn injury was induced by applying 38% HCl solution to the dorsum area of the mice for 1 min. AgNP-ointment was used twice a day in the treatment group.

### 3.3 Histological analysis

The closure of the burn injury was recorded using a digital camera on different days, and the surface area of the burn injury was measured with ImageJ software. The healing rate of burn injury was expressed as the change in a wound area relative to the original wound area. The mice were sacrificed at 1 and 4 days; their skin tissue was collected from the wound site and fixed in 4% buffered formaldehyde. Afterward, the skin tissue samples were embedded in paraffin, and 10 µm thick sections were obtained using a rotary microtome. Sections were stained by Hematoxylin and Eosin (H&E) and Toluidine Blue staining methods.

### 3.4 Reverse transcription-polymerase chain reaction analysis

After inflicting burn injury, the mice were euthanized by cervical dislocation on day 4. Tissue samples were collected from the dorsum area of the mice to isolate RNA for PCR analysis. Each tissue sample was homogenized in 1 ml of Trizol (Sigma Aldrich) reagent with 0.2 ml of chloroform (Chen et al., 1007). After centrifugation at 13,000 rpm at 4°C for 20 min, the supernatant containing RNA was transferred into a new vial and the RNA was precipitated with isopropanol. After an incubation period of 10 min and another centrifugation step of 15 min at 13,000 rpm, the supernatant was discarded. The pellet was washed with 1 ml of 70% ethanol and centrifuged for 15 min at 13,000 rpm. The supernatant was then discarded and the pellet dried. After adding 50 μl diethylpryocarbonate (DEPC)-treated water, the pellet was allowed to dissolve on ice for 10 min. The amount and purity of total RNA were determined using 260 and 280 nm absorbance measured by a spectrophotometer. cDNA library was developed with an RNA template using M-MLV RTase. Primer sequences used for PCR are listed in Table 1. The expression level of each gene was normalized with GAPDH. The ImageJ software (National Institutes of Health, Bethesda, MD) was used to conduct a quantitative analysis of PCR amplicons on digitized gel images.

**TABLE 1:** Chosen primer sequences.

Gene	Forward (F)/Reverse (R)	
TNF-λ	F	5′- CAT​CTT​CTC​AAA​ATT​CGA​GTG​ACA A-3′
	R	5′- TGG​GAG​TAG​ACA​AGG​TAC​AAC​CC -3′
IL-1β	F	5′- TGA​CGG​ACC​CCA​AAA​GAT​GA -3′
	R	5′- AAA​GAC​ACA​GGT​AGC​TGC​CA -3′
IL-6	F	5′-TGA​CAG​CCA​CTG​CCT​TCC​CTA​C-3′
	R	5′-CAA​TCA​GAA​TTG​CCA​TTG​CAC​AA-3′
IL-10	F	5′-GCA​CTG​CTA​TGT​TGC​CTG​CTC​TT-3′
	R	5′-GAG​CAT​GTG​GGT​CTG​GCT​GAC​T-3′
GAPDH	F	5′- CAT​GGC​CTT​CCG​TGT​TCC​TA -3′
	R	5′- ACT​TGG​CAG​GTT​TCT​CCA​GG -3′

### 3.5 Statistical analysis

All data expressed as the mean ± standard deviation (SD) and statistical significance were analyzed with a Student t-test, and a value of *p* < 0.05 was considered significant.

## 4 Results and discussion

### 4.1 Color change and UV-Vis spectroscopy analysis

**FIGURE 1:**
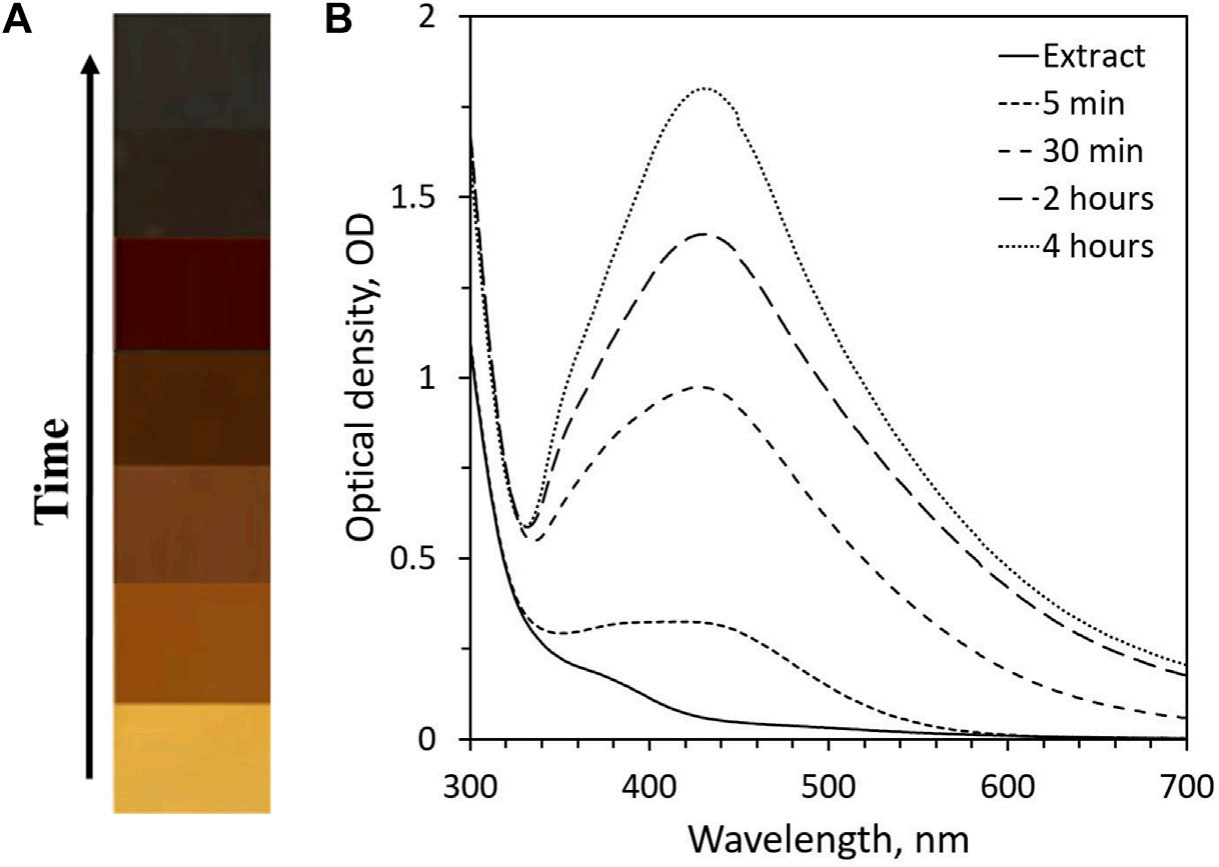
The reaction mixture containing AgNP synthesized with *Rhodiola rosea*: **(A)** color change; **(B)** UV-Vis spectra of AgNP as a function of time.

The presence of AgNP was first identified by color change as a function of time. The mixture turned brown immediately after adding plant extract to the AgNO_3_ solution and later changed to a darker shade (Figure 1A). The reaction mixture was observed from 5 min to 4 h. The change in color of the colloidal solution confirms the bioreduction of silver ions due to the plant extracts. The dark brown color of the colloidal solution occurs as a result of light absorbance in the visible region and collective oscillation of free electrons of AgNP, referred to as surface plasmon resonance (SPR). The color change from pale yellow to dark brown for the synthesized AgNP is in line with other published reports (Javed and Mashwani, 2020; UP et al., 2020; Vorobyova et al., 2020). UV-Vis spectroscopy analysis further confirmed the formation of the AgNP synthesized with *Rhodiola rosea*. The method is utilized in the preliminary stages to determine the size of AgNP, synthesis rate, yield, and stability by measuring the optical absorbance spectra of nanoparticles. The measurement results revealed the maximum absorbance at around 430 nm with a single narrow SPR peak, confirming the presence of spherical and monodisperse AgNPs (Figure 1B). An appearance of an SPR peak between 400 and 500 nm indicates the presence of synthesized silver nanoparticles (Sherin et al., 2020). The SPR peak value or wavelength provides approximate size information. Characterization on UV-Vis spectrophotometer showed a stable absorbance as a function of time and absence of peak shift that correlates to high yield and stability of AgNP. The flavonoids and phenolic compounds in the plant extract may be the main reducing and capping agents for forming AgNPs. Several studies reported that the flavonoid is the main compound responsible for reducing and capping AgNP (Alomar et al., 2020).

### 4.2 FTIR spectral analysis of the synthesized AgNP

**FIGURE 2:**
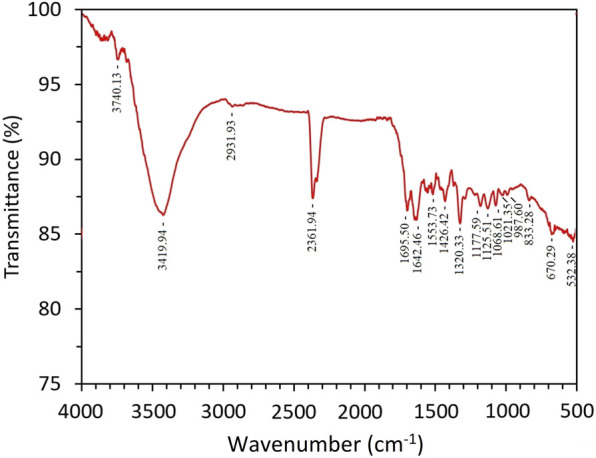
Fourier transform spectra of AgNP synthesized with *Rhodiola rosea.*

The surface chemistry, functional groups, and residues adhered to the nanoparticles can be studied using the FTIR technique (Nayaka et al., 2020). AgNPs were synthesized with *Rhodiola rosea*. The synthesized AgNP was analyzed by FTIR spectroscopy to study the functional groups responsible for reducing and capping nanoparticles (Figure 2). FTIR analysis results further prove the crucial role of biologically active compounds in the plant extract in reducing and capping the AgNP. The functional groups detected in the AgNP is as follows, the bands were identified at 532.38, 670.29, 833.28, 987.60, 1021.35, 1068.61, 1125.51, 1177.59, 1320.33, 1426.42, 1553.73, 1642.46, 1695.50, 2361.94, 2931.93, 3419.94, and 3740.13 cm^−1^. The broadband at 3419.94 cm^−1^ is related to O-H stretching, which may refer to phenols, alcohols, or the NH functional group of amides or amine (Hafeez et al., 2021). Other bands found in AgNP, such as 1426.42, 1553.73, 1642.46, 1695.50, 2361.94, and 2931.93 cm^−1^ are assigned to S=H sulfur ester, C–O stretch, C=C stretch, C=O stretch, P–H phosphine and C–H stretch respectively.

### 4.3 Surface charge and stability of the synthesized AgNP

Zeta Potential is an essential parameter for understanding the synthesized nanoparticles’ stability and surface charge in the colloid system. Nanoparticles can be considered stable if the zeta potential values are above +30 mV or below −30 mV; this is due to the electrostatic repulsion between nanoparticles in suspension (Kumar et al., 2019). The silver nanoparticle synthesized with the medicinal plant *Rhodiola rosea* revealed a zeta potential of −68.38 ± 3.4 mV, indicating strong stability and a low tendency to aggregation (Table 2). The negative zeta potential implies that the synthesized silver nanoparticle is capped and stabilized by an electronegative compound, which may be the biomolecules within the *Rhodiola rosea* plant.

**TABLE 2:** PCCS, EDX, and Zeta potential values.

PCCS (nm)			EDX (%)				Zeta potential (mV)
X_90_ = 67.5	SMD = 57.7	VMD = 58.4	Ag = 81.9	Cl = 2.6	*p* = 13.8	K = 1.7	−68.38 ± 3.4

### 4.4 XRD, PCCS, SEM, and EDX analysis

**FIGURE 3:**
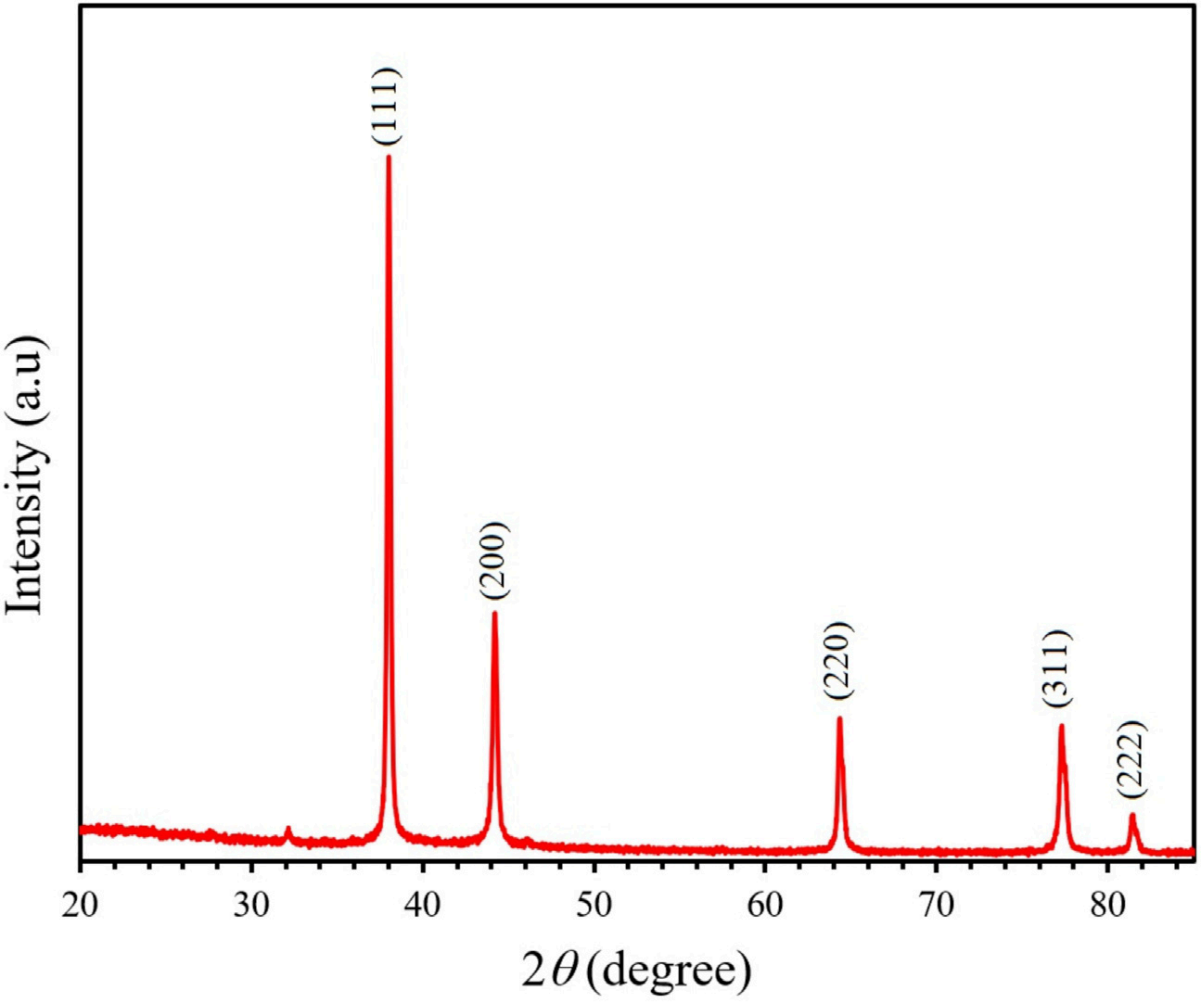
XRD spectra of the synthesized AgNP. Peaks appeared at 111, 200, 220, 311, and 222 plane lattice.

**FIGURE 4:**
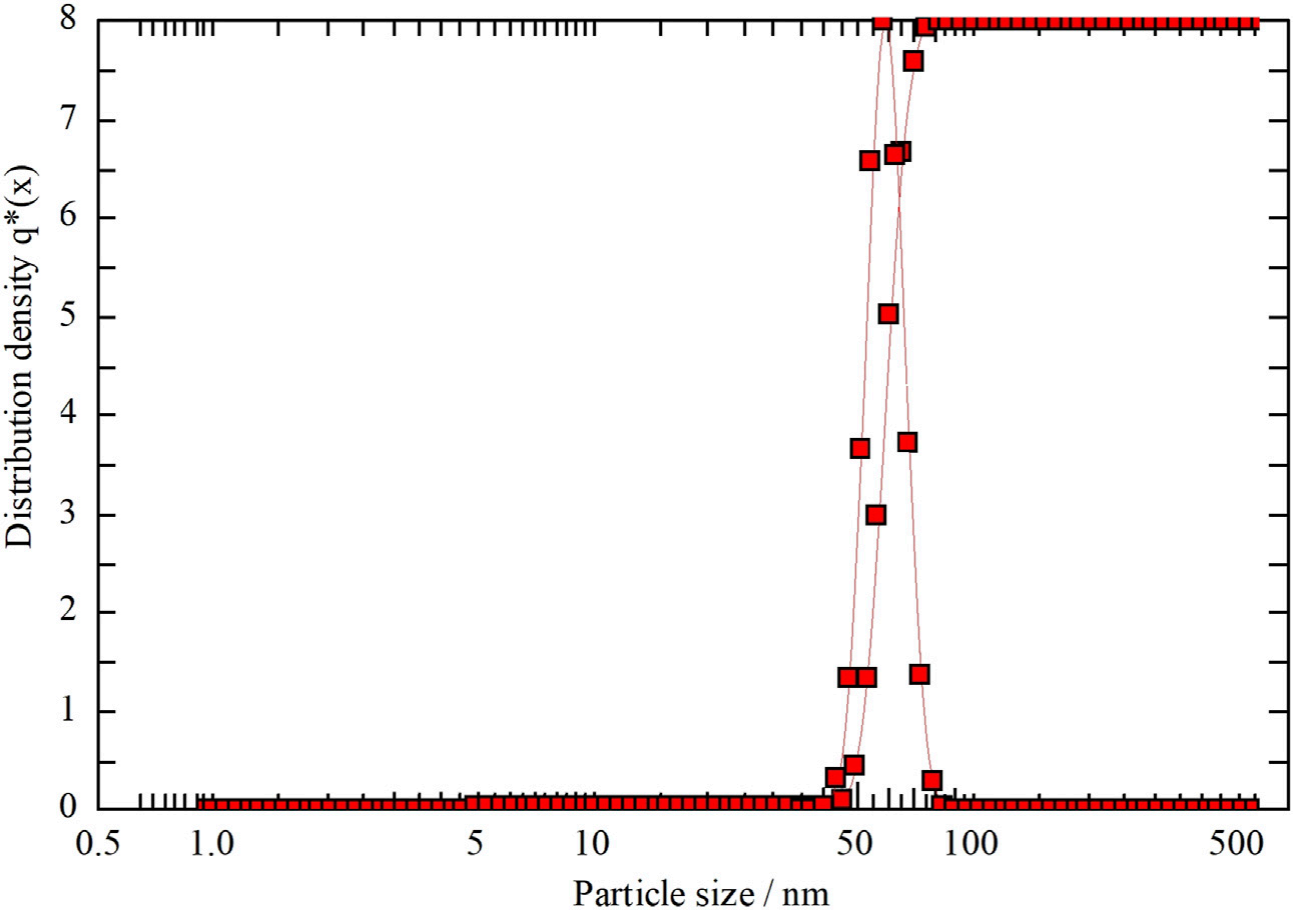
PCCS analysis of the nanoparticle distribution of the synthesized by *Rhodiola rosea*.

**FIGURE 5:**
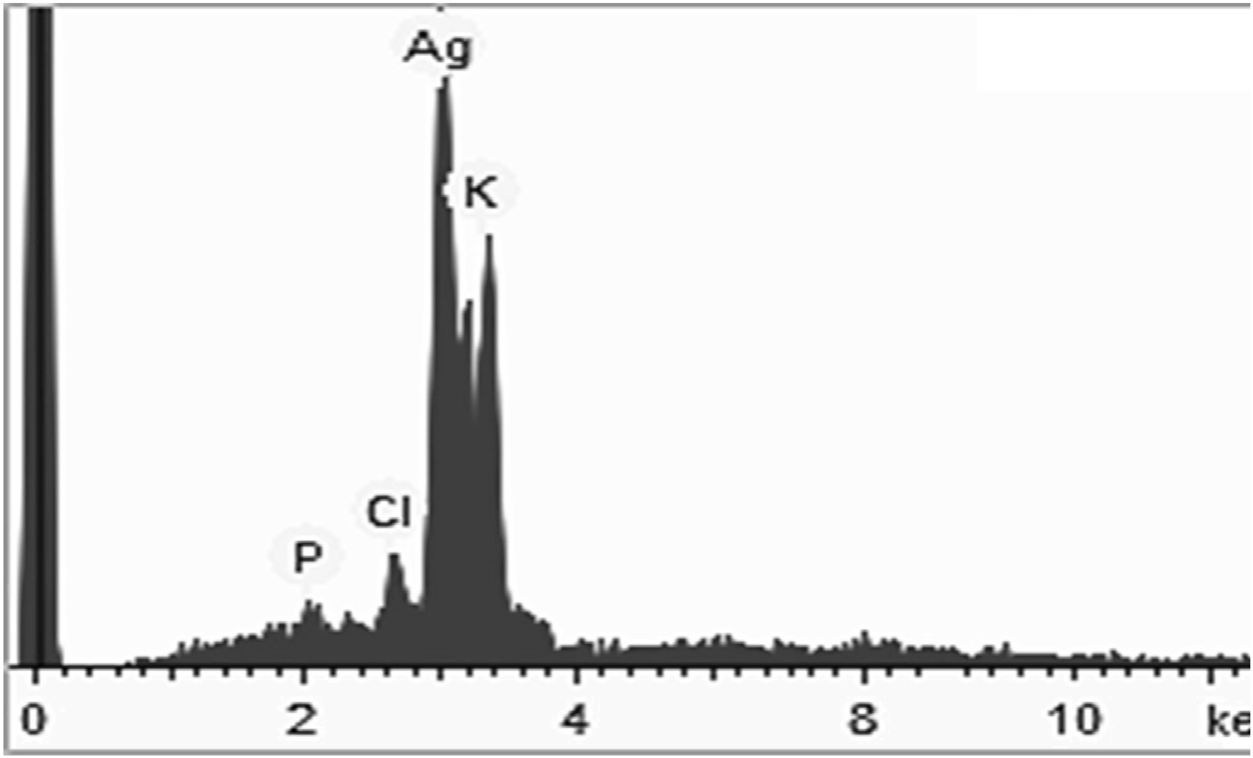
EDX analysis of AgNP synthesized by *Rhodiola rosea*.

**FIGURE 6:**
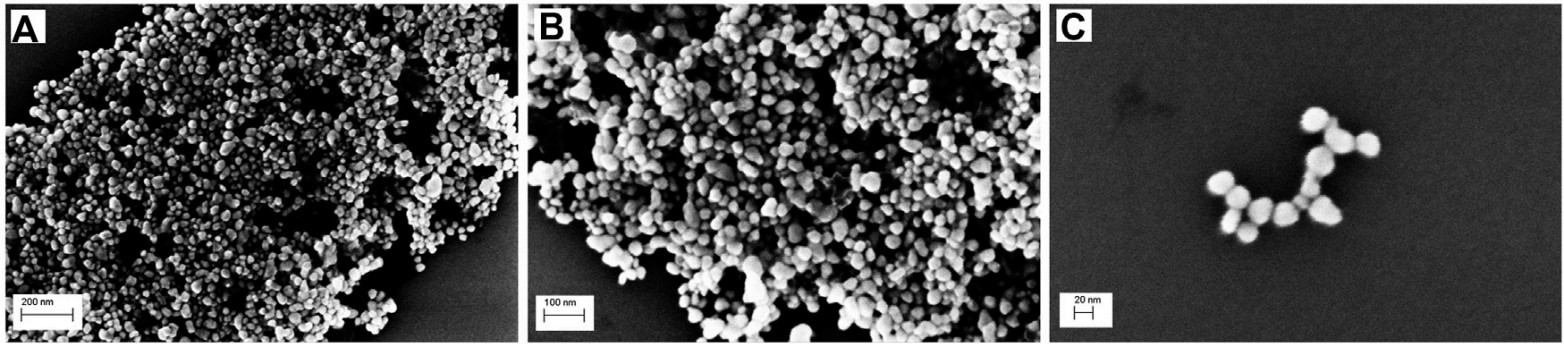
SEM image of the biosynthesized AgNP with different magnifications: **(A)** 200 nm, **(B)** 100 nm, **(C)** 20 nm.

The crystalline nature of the synthesized AgNP with *Rhodiola rosea* was studied and carried out by X-Ray crystallography. The XRD pattern of the nanoparticles was analyzed with an XRD instrument and shown in Figure 3. Bragg reflection of the 2*θ* peaks was observed at 20° to 90° and corresponded to (111), (200), (220), (311), and (222) plane lattice, which can be indexed to the face-centered cubic crystal nature of the silver with crystallite size of 23 nm. The result matches the reported reference value of ICSD No. 064994. PCCS is a technique that measures the average nanoparticle size (grain size) based on the Brownian motion. In Figure 4, the average particle size of AgNP was approximately X_90_ = 67.5 nm, SMD = 57.7 nm, and VMD = 58.4 nm. The difference between PCCS and XRD analysis lies in the measurement method of the particle. Scanning Electron Microscope (SEM) and Energy Dispersive X-Ray Spectroscopy (EDX) characterized the morphological and elemental analysis. EDX analysis revealed the elemental composition of the synthesized AgNP (Figure 5), which included 81.9% silver, 2.6% chlorine, 13.8% phosphorus, and 1.7% potassium elements (Table 2). In addition, the nitrogen element was not detected in the sample, implying no presence of trace ions from AgNO_3_. And the SEM imaging has confirmed the presence and spherical shape of the synthesized AgNP and monodisperse size distribution of ∼23 nm (Figure 6).

### 4.5 Evaluation of antibacterial activity

**FIGURE 7:**
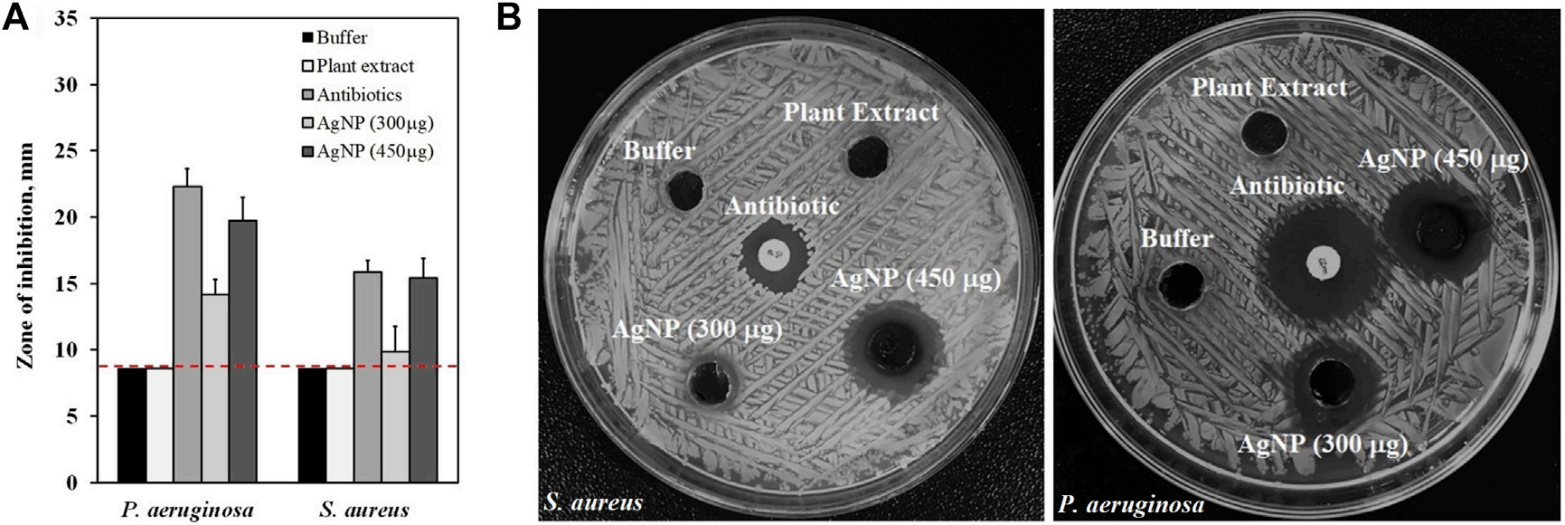
Antibacterial activity of the synthesized AgNP against *S. aureus* and *P. aeruginosa*: **(A)** diameter of the inhibition zone with standard deviation, **(B)** Images of agar plates loaded with AgNP (300 and 450 μg/ml), Antibiotics (erythromycin and penicillin G), Plant Extract and HEPES buffer. Dotted red lines represent level of no activity.

The antibacterial activity of AgNP (300 μg/ml and 450 μg/ml) was evaluated against *P. aeruginosa* and *S. aureus* bacteria. The erythromycin and penicillin G were selected as the positive control, and the plant extract and HEPES buffer as the negative control group of the study. Figure 7A shows the diameter of the inhibition zone of AgNP, the results reveal antibacterial activity against both *P. aeruginosa* and *S. aureus* bacteria. The plant extract and HEPES buffer did not show antibacterial activity on the studied bacteria. In addition, *P. aeruginosa* bacteria were more susceptible to the synthesized AgNP compared to *S. aureus* bacteria (Figure 7B), this may be due to the difference in bacterial cell wall structures (Vijayan et al., 2018). However, the AgNP synthesized with *Rhodiola rosea* exhibited an effective activity against both pathogenic bacteria. Currently, the antibacterial effect of AgNP is not clearly understood yet. It has been postulated that the antibacterial mechanism of action is a result of a simultaneous process (Hambardzumyan et al., 2020): 1) penetration of AgNP in the cell wall of bacteria leading to cellular content leakage (Rajesh et al., 2020); 2) silver ion release (Vorobyova et al., 2020); reaction with sulfhydryl groups inside the cytosol (Mickymaray, 2019).

### 4.6 Evaluation of antioxidant activity

#### 4.6.1 Measurement of DPPH scavenging activity

**FIGURE 8:**
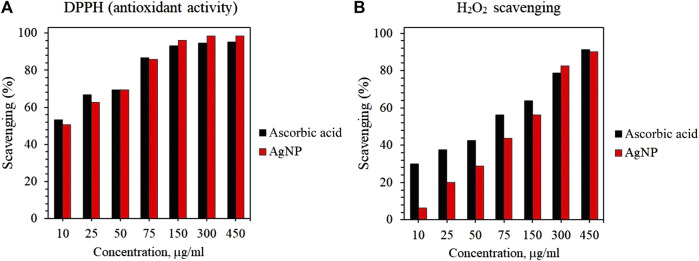
Antioxidant activity of AgNP: **(A)** DPPH free radical scavenging activity of AgNPs; **(B)** H_2_O_2_ scavenging activity of AgNPs.

The antioxidant activity of AgNP was evaluated using DPPH free radical scavenging assay. The DPPH is a well-known compound for its ability to accept hydrogen or electrons (Al-Shmgani et al., 2017). The DPPH scavenging activity of AgNP was first observed through a color change, the control with DPPH only did not show any color change. Figure 8A shows that the synthesized AgNP exhibited inhibition of 50.66%–98.53% in a dose-dependent manner at concentrations of 10–450 μg/ml. In addition, AgNP synthesized by *Rhodiola rosea* showed slightly higher scavenging activity at concentrations of 150–450 μg/ml compared to standard ascorbic acid. The free radical scavenging activity of AgNP may be attributed to the biomolecules adhered to the surface of the nanoparticles, thereby increasing the surface area for the antioxidant activity (Lateef et al., 2016).

#### 4.6.2 H_2_O_2_ scavenging activity

The H_2_O_2_ scavenging activity of AgNPs was evaluated and the results are shown in Figure 8B. First, the scavenging activity was confirmed by the color change of the solution after the addition of AgNP. Similar to DPPH scavenging activity, AgNP displayed H_2_O_2_ scavenging activity in a dose-dependent manner and showed similar efficacy to the standard ascorbic acid. However, the H_2_O_2_ scavenging of AgNPs was lower compared to DPPH activity. Within the tested AgNPs concentrations, the highest scavenging activities of AgNPs and ascorbic acid were detected at 450 μg/ml. The results of the DPPH and H_2_O_2_ scavenging activity suggest the possible application of AgNP as an antioxidant agent.

### 4.7 Cytokine expression of burn wound healing

**FIGURE 9:**
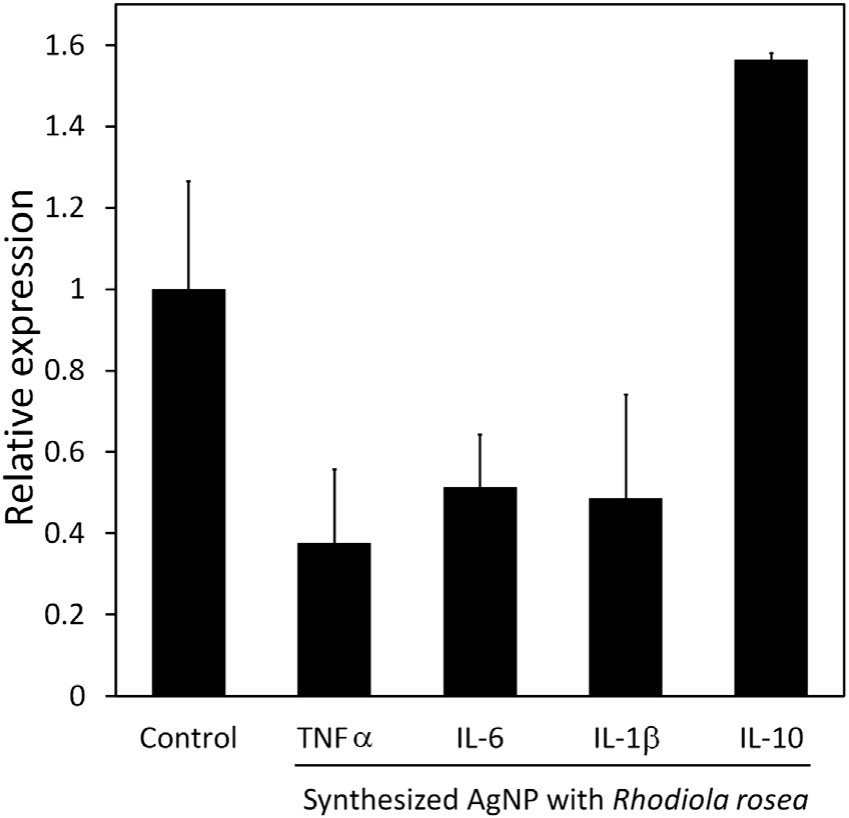
Cytokine expression of murine model of treatment and control group. PCR analysis of inflammation-related genes, TNF-α, IL-6, IL-1β, and IL-10. Expression levels were normalized to GAPDH. Data are presented as mean SEM. Three independent experiments were carried out.

After post-burn injury of the murine model, the TNF-α, IL-1β, and IL-6 expression levels in the treatment group were significantly less than in the control group (Figure 9). On the other hand, there was an elevation of IL-10 expression in the treatment group. The anti-inflammatory cytokine IL-10 prevents excessive inflammation during the wound healing phase by inhibiting the synthesis of pro-inflammatory cytokines, including IL-6, IL-1, and TNF-α. The down-regulation of pro-inflammatory cytokine IL-6 leads to less macrophage and neutrophil recruitment on the wound site, thus fewer cytokine releases that may prolong the inflammatory phase, thereby delaying the wound healing. Additionally, TNF-α is considered the first responder to inflammation, and the cytokine activates keratinocytes and macrophages to release ROS, iNOS, and IL-1β. Overall, biosynthesized AgNP revealed that it could suppress pro-inflammatory cytokines, such as TNF-α, IL-1β, and IL-6, and promote the expression of anti-inflammatory cytokine IL-10 on burn injury.

### 4.8 Macroscopic and histological evaluation of burn wound

**FIGURE 10:**
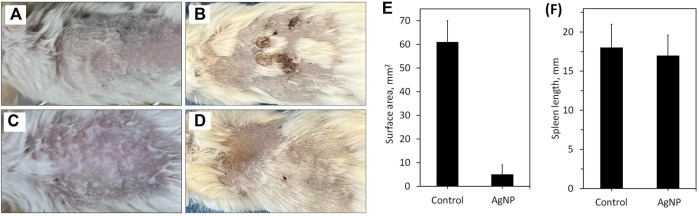
Macroscopic evaluation of burn injury on mice: **(A)** 1 day after burn injury in control group; **(B)** 4 days after burn injury in control group; **(C)** 1 day after burn injury in treatment group; **(D)** 4 days after burn injury in the treatment group; **(E)** changes in the surface area after experiment at 4 days; **(F)** changes in the spleen length after experiment at 4 days.

**FIGURE 11:**
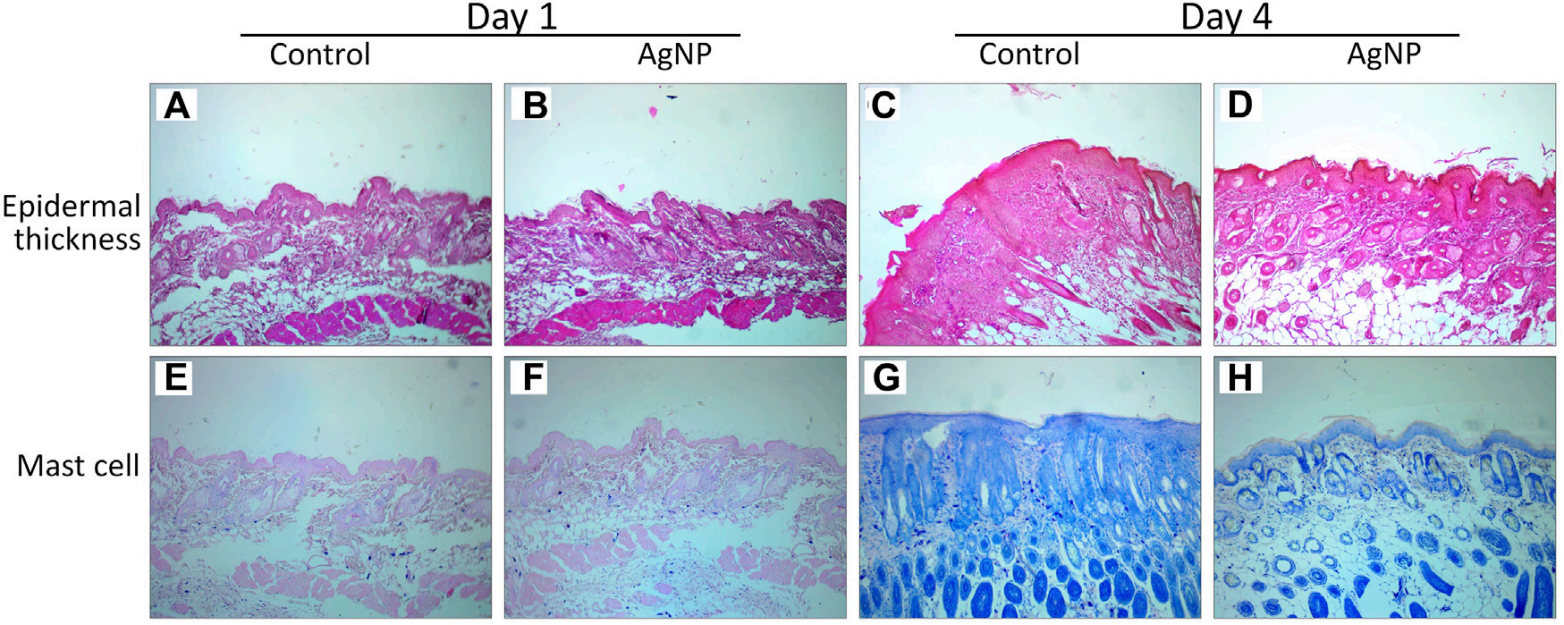
Effect of AgNP on histologic features of the wound at 1 and 4 days: **(A,C)** H&E staining on control group; **(B,D)** H&E staining on treatment group; **(E,G)** Toluidine blue staining on control group; **(F,H)** Toluidine staining on treatment group.

The inflammatory response is an essential part of the wound healing process and one of the significant causes of burn complications. The effect of AgNP-ointment on burn wound healing was studied using macroscopic evaluation of burn wound surface area and changes in spleen length using digital photographs (Figure 10). The burn wound healing is shown in Figure 10; there was a significant difference in the closure of the burn wound surface area between the control and treatment groups by 61 ± 9 mm^2^ and 5 ± 4 mm^2^ respectively (Figure 10E). All tested mice in the control group developed scabs on the wound site, while mice in the treatment exhibited newborn hair follicles on the wound site (Figures 10A–D). Overall, the macroscopic appearance and closure of the burn wound area suggest accelerated wound healing with the application of AgNP-ointment. There were no significant changes in spleen length between the control and treatment groups which signifies that AgNP-ointment did not exert adverse systemic toxicity in the spleen (Figure 10F). The regeneration of the epithelial layer and mast cell migration of the control and treatment groups was investigated using H&E, Toluidine Blue staining (Figure 11). The re-epithelization process involves the thickening to thinning of the epithelial layer, where cells evolve from proliferation to differentiation until skin thickness is normalized (Wang et al., 2020). In the control group, a thick epithelial layer is observed after inflicting burn injury; in contrast, the AgNP-treatment group exhibited thinning of the epithelial layer (Figures 11A–D). Subsequently, Toluidine Blue staining also revealed pronounced mast cell migration in the control group; in comparison, mice treated with AgNP-ointment were infiltrated with fewer mast cells on the wound site (Figures 11E–H).

## 5 Conclusion

In the current study, AgNP was synthesized by the medicinal plant *Rhodiola rosea* using a safe, non-toxic and eco-friendly approach. The synthesized AgNP were subjected to different characterization methods, such as UV-Vis spectroscopy, FTIR, XRD, PCCS, SEM, and EDX. The synthesized AgNP exhibited an absorbance at 430 nm which confirmed the formation of nanoparticles, and the surface charge of −68.38 ± 3.4 mV revealed high stability of nanoparticles. The XRD and PCCS confirmed a face-centered cubic structure with a crystallite size of 23 nm and an average grain size of 67.5 nm. The synthesized AgNP exhibited both antioxidant and antibacterial activity. Moreover, the wound-healing effect of AgNP was evaluated by preparing AgNP-loaded ointment, thereafter its efficacy was studied on burn injury. AgNP-loaded ointment regulated inflammatory cytokines involved in the wound healing process. The synthesized AgNP may be used as a possible candidate for further application in the biomedical field.

## Data Availability

The datasets presented in this study can be found in online repositories. The names of the repository/repositories and accession number(s) can be found below: https://www.ncbi.nlm.nih.gov/, XM_036165840.1 https://www.ncbi.nlm.nih.gov/, XM_006498795.5 https://www.ncbi.nlm.nih.gov/, NM_013693.3.
